# Bismuth-induced effects on optical, lattice vibrational, and structural properties of bulk GaAsBi alloys

**DOI:** 10.1186/1556-276X-9-119

**Published:** 2014-03-14

**Authors:** Fahrettin Sarcan, Ömer Dönmez, Kamuran Kara, Ayse Erol, Elif Akalın, Mehmet Çetin Arıkan, Hajer Makhloufi, Alexandre Arnoult, Chantal Fontaine

**Affiliations:** 1 Department of Physics, Faculty of Science, Istanbul University, Vezneciler, Istanbul 34134, Turkey; 2 CNRS, LAAS, 7 Avenue du Colonel Roche, Toulouse 31400, France; 3 University of Toulouse, Toulouse 31400, France

**Keywords:** GaAsBi, Dilute bismide, S-shape, Varshni’s law

## Abstract

Bulk GaAs_1 - *x*_Bi_*x*_/GaAs alloys with various bismuth compositions are studied using power- and temperature-dependent photoluminescence (PL), Raman scattering, and atomic force microscopy (AFM). PL measurements exhibit that the bandgap of the alloy decreases with increasing bismuth composition. Moreover, PL peak energy and PL characteristic are found to be excitation intensity dependent. The PL signal is detectable below 150 K at low excitation intensities, but quenches at higher temperatures. As excitation intensity is increased, PL can be observable at room temperature and PL peak energy blueshifts. The quenching temperature of the PL signal tends to shift to higher temperatures with increasing bismuth composition, giving rise to an increase in Bi-related localization energy of disorders. The composition dependence of the PL is also found to be power dependent, changing from about 63 to 87 meV/Bi% as excitation intensity is increased. In addition, S-shaped temperature dependence at low excitation intensities is observed, a well-known signature of localized levels above valence band. Applying Varshni’s law to the temperature dependence of the PL peak energy, the concentration dependence of Debye temperature (*β*) and thermal expansion coefficient (*α*) are determined. AFM observations show that bismuth islands are randomly distributed on the surface and the diameter of the islands tends to increase with increasing bismuth composition. Raman scattering spectra show that incorporation of Bi into GaAs causes a new feature at around 185 cm^-1^ with slightly increasing Raman intensity as the Bi concentration increases. A broad feature located between 210 and 250 cm^-1^ is also observed and its intensity increases with increasing Bi content. Furthermore, the forbidden transverse optical (TO) mode becomes more pronounced for the samples with higher bismuth composition, which can be attributed to the effect of Bi-induced disorders on crystal symmetry.

**PACS:**

78.55Cr 78.55-m 78.20-e 78.30-j

## Background

Recently, it has been realized that the incorporation of a small percentage of bismuth (Bi) into GaAs results in a drastic decrease of bandgap energy, thus making GaAsBi a promising alloy for device applications operating in the near-infrared region [1,2]. Additionally, spin-orbit splitting energy has been found to increase with increasing Bi content, thereby also establishing GaAsBi as a promising alloy for spintronic applications [3]. GaAsBi can also be considered as an alternative to dilute nitrides whose electron mobility is drastically affected by the influence of nitrogen on the conduction band and nitrogen-induced defects [4]. Since the localized level of bismuth in GaAs only restructures the valence band, it is predictable that the electron mobility is not affected by bismuth content [5]. As a result, incorporation of Bi into GaAs leads to a desirable red shift of bandgap energy, while electron mobility remains unaffected [6]. However, the incorporation of bismuth into the III-V lattice requires low-temperature growth conditions, thus causing formation of the defects as previously experienced in all the members of the highly mismatched alloys [2]. Hence, the optimization of GaAsBi growth conditions to enhance the optical and electrical quality of the alloy is still a challenge. An in-depth study of the fundamental properties of GaAsBi is strongly needed in order to explore its potential for commercial usage [7].

In the present work, we have studied molecular beam epitaxy (MBE)-grown bulk GaAsBi/GaAs samples with various bismuth compositions using temperature- and intensity-dependent photoluminescence (PL), atomic force microscopy (AFM), and Raman spectroscopy.

## Methods

GaAsBi epilayers were grown on semi-insulating GaAs substrates using MBE at 360°C to 390°C, while keeping the As/Bi atomic species ratio close to unity. The thickness of the epilayers was in the range of 200 to 250 nm. The temperature was first calibrated, thanks to a bandgap thermometry (BandIT; k-Space Associates, Inc., Dexter, MI USA). The error on growth temperature was estimated to be ±5°. Since bismuth incorporation is known to be highly dependent on substrate temperature, X-ray diffraction was used to accurately determine the bismuth content in the epilayers. All layers were found to be elastically strained. PL measurements were carried out between 40 and 300 K using the 514.5-nm line of an Ar^+^ laser as an excitation source. The PL signal was dispersed with a 0.5-m high-resolution monochromator and detected using nitrogen-cooled InGaAs photomultiplier. The surface morphology of the samples was monitored and analyzed using AFM in tapping mode. A Jasco NRS 3100 Raman spectrometer (Jasco Corporation, Tokyo, Japan) equipped with a CCD detector was used for recording the micro-Raman spectrum with a diode laser operating at 532 nm as an excitation source. All measurements were performed at room temperature in a back-scattering geometry. The power of laser source was kept as low as a few milliwatts.

## Results and discussion

**Figure 1:**
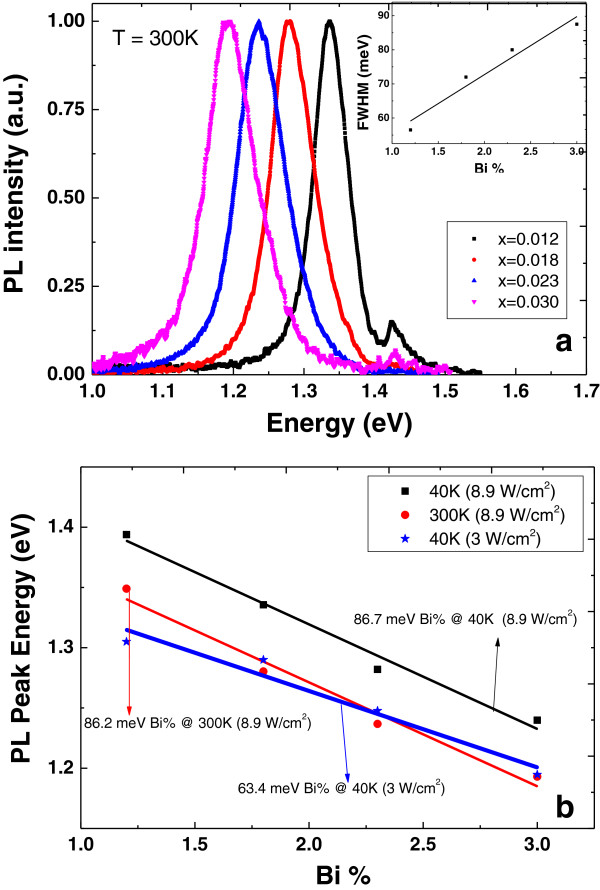
**PL spectra and composition dependence of PL peak. (a)** PL spectra for the samples. The inset shows Bi composition dependence of FWHM. **(b)** PL peak energy versus Bi composition at different excitation intensities.

Figure 1a shows that the bandgap of GaAsBi red shifts with increasing Bi composition due to the stronger interaction between valence band and localized Bi level [8]. The amount of the red shift is intensity dependent as shown in Figure 1b. At low intensity (3 W/cm^2^), the compositional dependence of the PL peak energy is found to be 63 meV/Bi% at 40 K. At the same temperature (*T* = 40 K), with higher excitation intensity, the amount of the red shift of the PL peak energy increases to 87 meV/Bi%. This observation indicates a contradiction in the existing literature regarding the compositional dependence value of GaAsBi at low temperatures [9]. The inset of Figure 1a shows the compositional dependence of full width at half maximum (FWHM) of PL spectra at 300 K. It increases monotonically with rising Bi composition due to the escalation of Bi spatial fluctuations in the alloy composition. The fact that the observed FWHM value is about three to four times larger than that of GaAs (20 to 25 meV) is an indication of optical degradation due to the increased Bi-related density of defects [10].

**Figure 2:**
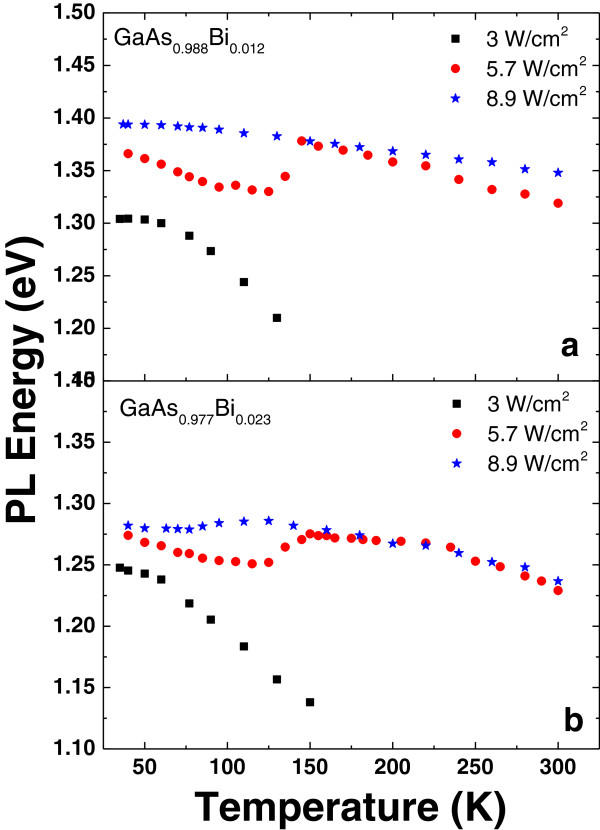
**Temperature dependence of PL peak energy at various excitation intensities. (a)** GaAs_0.988_Bi_0.012_. **(b)** GaAs_0.977_Bi_0.023_.

It has been demonstrated that low-temperature PL in these alloys is usually dominated by localized excitons in the states that are formed by the clusters and/or alloy fluctuations of the highly mismatched atom in the alloy [11]. We have observed the similar characteristic as seen in Figure 2. At low temperatures (*T* ≤ 150 K), PL originates from localized excitons at low excitation intensities. PL intensity is highly temperature-sensitive and quenches below room temperature, demonstrating that there is no contribution from free exciton transition at this excitation intensity. We have observed that as Bi concentration increases, the quenching temperature of the PL signal tends to increase slightly in the detection limit of the PL setup. Figure 2 shows that for samples GaAs_0.988_Bi_0.012_ and GaAs_0.977_Bi_0.023_, the room temperature PL can be observed at higher excitation powers.

The temperature dependence of PL peak energy at low excitation intensities has a pronounced S-shape characteristic (see Figure 2), which is also typical of the highly mismatched alloys [12]. The S-shaped temperature dependence also originates from the PL of localized states in GaAsBi at low temperatures. When the temperature exceeds 150 K, the PL spectrum is dominated by free exciton emission. With increasing excitation intensity due to the filling of the localized levels, the S-shaped region moves towards lower temperatures (see Figure 2b) and then disappears at higher intensities. This observation can be explained by filling all localized states with photo-generated carriers under high excitation power, thus causing a band-to-band transition of free carriers. Consequently, the PL peak energy blueshifts at low temperatures (≤150 K) under higher excitation intensities and sheds light on the observed discrepancy of the red shift values per Bi% at different excitation intensities (Figure 1b).

**Figure 3:**
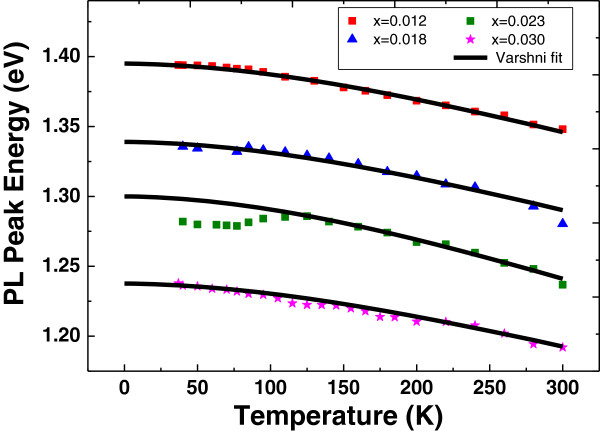
**Temperature dependence of PL peak energy for the samples with various bismuth contents.** Solid lines represent Varshni’s fit.

Figure 3 shows the temperature-induced shift of the bandgap energy at an excitation intensity of 8.9 W/cm^2^. We have observed that the temperature-induced shift of the bandgap energy between 40 and 300 K is smaller than that of GaInNAs with similar nitrogen content [13]. It is worth noting that even at this high excitation intensity (8.9 W/cm^2^), the sample GaAs_0.977_Bi_0.023_ still maintains S-shaped temperature dependence, and for this reason, true temperature dependence of the bandgap energy for GaAs_0.977_Bi_0.023_ is debatable.

**Figure 4:**
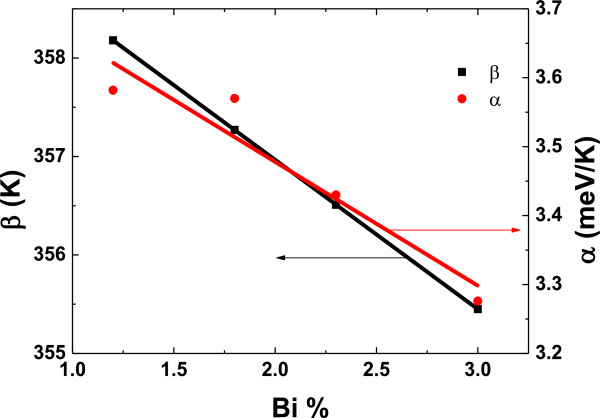
Varshni’s parameters, α and β, versus Bi composition.

The temperature dependence of PL peak energy is fitted using the semi-empirical Varshni’s formula:

(1) $$E_{g}\left(T\right)=E_{0}-\frac{\alpha T^{2}}{\beta +T}$$

where *E*_0_ is the bandgap energy at 0 K, *α* is thermal expansion coefficient, and *β* is Debye temperature [14]. The Debye temperature is calculated for GaBi using the elastic constants of the zinc-blende phase [15], and Debye temperature of GaAs is taken as 360 K [16]. Composition-dependent Debye temperature of GaAs_1 - *x*_Bi_*x*_ alloy is determined using Vegards law:

(2) $$\beta_{{\text{GaAs}}_{1-x}{\mathrm{Bi}}_{x}}=\beta_{\mathrm{GaBi}}\;x+\beta_{\text{GaAs}}\left(1-x\right)$$

and thermal expansion coefficient is used as a fitting parameter. Figure 4 shows the compositional dependence of Varshni’s parameters. The elastic constants of GaAs and GaBi used in Equation 2 are tabulated in Table 1. As Bi concentration increases, the lattice constant of GaAsBi increases, causing a decrease in Debye temperature. Since elastic constants of GaAs are larger than those of GaBi (given in Table 1), the thermal expansion coefficient decreases with increasing Bi concentration.

**Table 1:** **Elastic constants of GaAs and GaBi for zinc-blende phase**[15]

	***C***_**11 **_**(GPa)**	***C***_**12 **_**(GPa)**	***C***_**14 **_**(GPa)**
GaAs	1.242	0.514	0.634
GaBi	0.730	0.327	0.363

**Figure 5:**
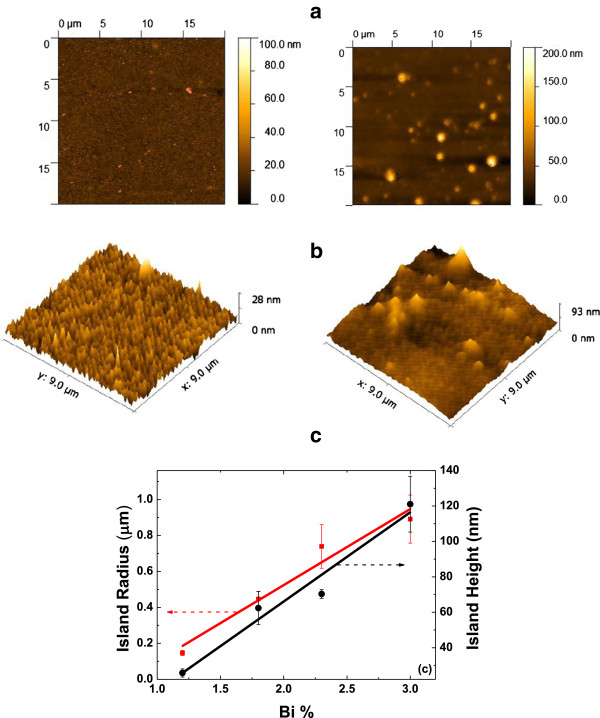
**2D and 3D AFM images of the samples and comparison. (a)** GaAs_0.988_Bi_0.012_ and **(b)** GaAs_0.97_Bi_0.03_. **(c)** Comparison of radius and height of islands versus Bi concentration.

The surface morphology of the samples is observed using AFM. As seen in Figure 5, for the samples GaAs_0.988_Bi_0.012_ and GaAs_0.97_Bi_0.03_, the surface of the samples has randomly distributed defects and size of the defects is observed to be larger with increasing Bi content.

The bismuth atom is larger in size and has lower vapor pressure in comparison to gallium and arsenic. For this reason, it has a strong tendency to segregate at the surface during GaAsBi growth [17]. It was claimed that bismuth islands could also easily nucleate on the surface [18]. Here, since near-stoichiometric conditions have been used for MBE growth of these layers, the same origin can be proposed as As/Ga ratio was kept slightly higher than the unity during growth. This concludes that the observed islands should be bismuth-related. Further work is clearly to ascertain the origin of these defects.

**Figure 6:**
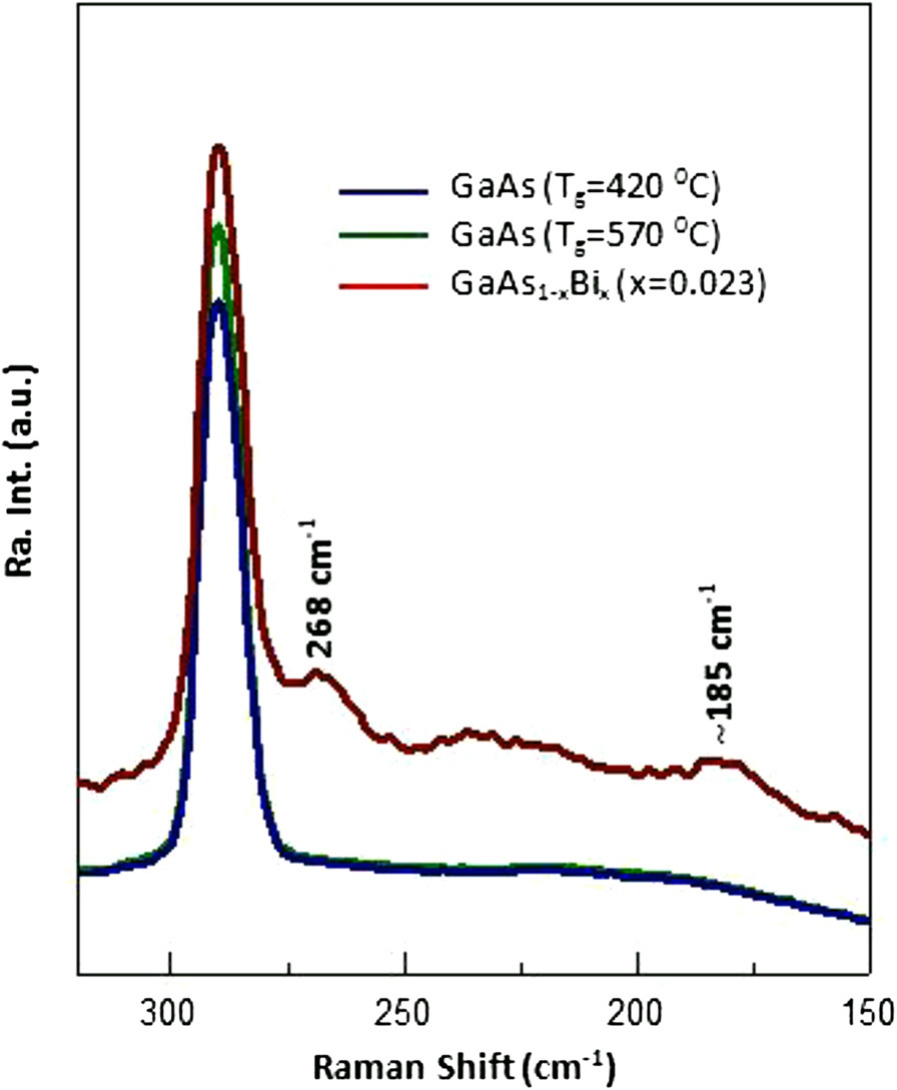
**Raman spectra of GaAs grown at low (420°C) and high temperature (570°C) and GaAs**_**0.977**_**Bi**_**0.023**_**.**

Figure 6 shows a comparison of Raman spectra for low-temperature (420°C), high-temperature growth (570°C) of GaAs, and GaAs_0.977_Bi_0.023_ samples in order to determine the origin of the vibrational peaks. We have not observed any significant differences in the Raman spectra of low- and high-temperature-grown GaAs samples. Both samples have longitudinal optical (LO) mode. As seen in Figure 6, for GaAs_0.977_Bi_0.023_, new features at about 185 and 268 cm^-1^ together with a broad one located between 210 and 250 cm^-1^ are observed. Verma et al*.* also observed the broad feature in the same spectral region and assigned it to As clusters because of the decrease in crystal quality [19]. On the other hand, Seong et al. also observed this broad peak in the same spectral range and assigned three features in this region at approximately 214 cm^-1^ (GaBi-related mode - Bi_As_), approximately 230 cm^-1^ (GaAs LA(X)), and 240 cm^-1^ (GaAs LO(L)). It was suggested that the observation of these forbidden modes was due to the Bi-induced mixing of GaAs valence band [20]. The presence of arsenic clusters in such an alloy is quite unexpected since bismuth plays a role as a surfactant during growth process, leading to delay in the incorporation of arsenic atoms [21-24]. This should favor the growth of layers with lower density of the arsenic point defects. Therefore, we believe that the proposal of Tixier et al*.* and Seong et al*.* related to Bi-induced mixing in valence band is more suitable to explain the origin of broad band.

**Figure 7:**
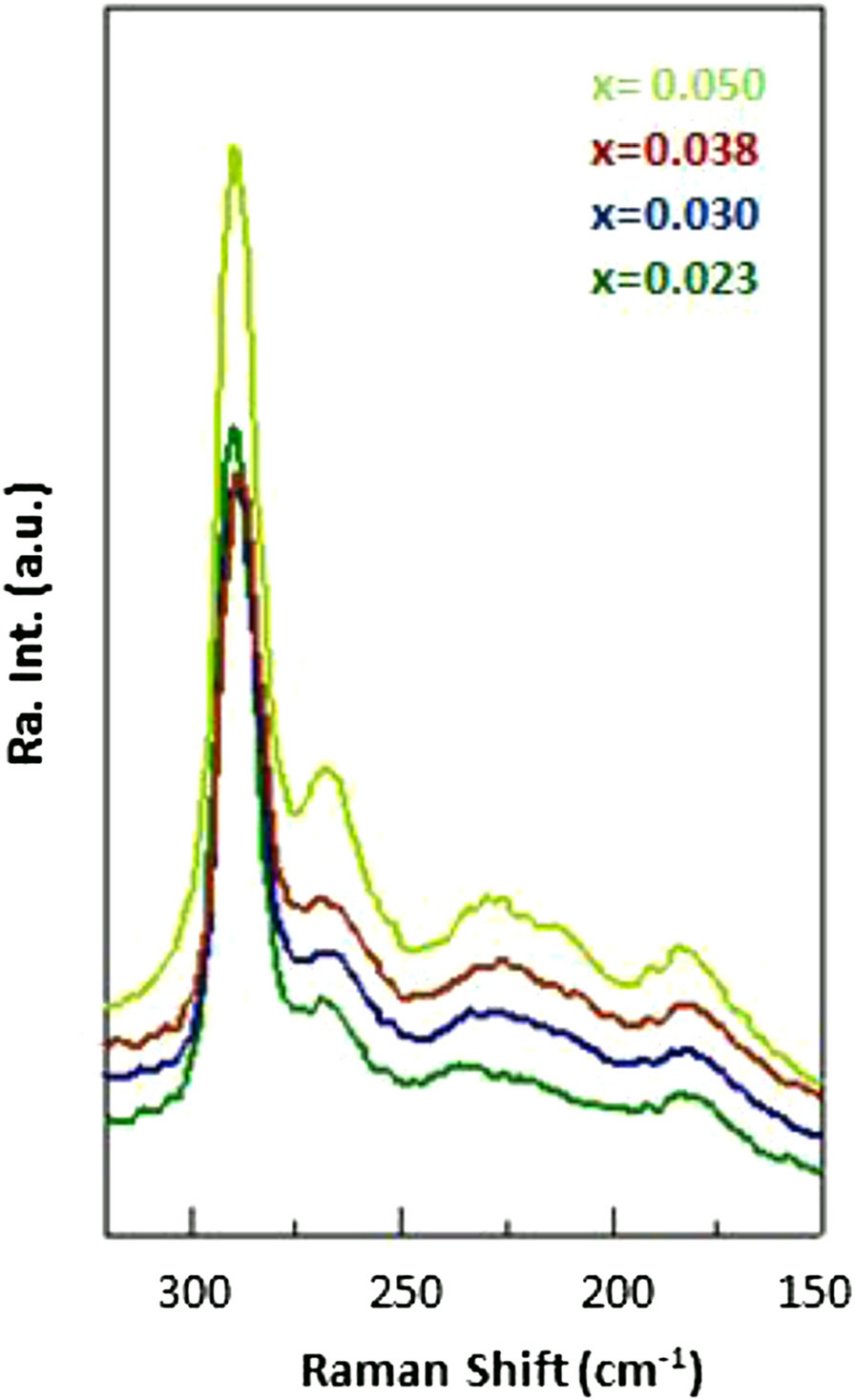
**Raman spectra of GaAs**_**1 - *x***_**Bi**_***x***_**(*x*** **= 5.0%, 3.8%, 3.0%, and 2.3%).**

As seen in Figure 7, with increasing bismuth content, the transverse optical (TO) mode, which is forbidden if the crystal has a perfect zinc-blende structure, becomes more pronounced. In addition, the intensity of the broad feature increases with increasing Bi content. The peak at approximately 185 cm^-1^ also slightly strengthens. Since it is not seen in GaAs, the Raman feature at 185 cm^-1^ can be attributed to the Bi-induced phonon mode. The intensity of this feature is much smaller than that of LO (GaAs), because the content of the Bi in the alloy is negligibly small compared to gallium and arsenic. The observation of increasing Raman intensity of the both TO mode and the broad feature at 210 to 250 cm^-1^ is an indication of the degradation of the crystal quality with increasing Bi concentration.

## Conclusions

The effect of Bi composition on optical, lattice vibrational, and structural properties of bulk GaAs_1 - *x*_Bi_*x*_/GaAs alloys was investigated. The compositional dependence of the bandgap was found to be excitation-intensity dependent, which is attributed to the presence of localized states. PL emission at low excitation intensities was observable at low temperatures, indicating that PL originates from localized excitons. On the other hand, at higher excitation intensities, the reason for the observation of the room temperature PL was due to the contribution of free excitons. The temperature-induced shift of the bandgap energy was found to be lower than those of classical III-V alloys and dilute nitrides. The use of Varshni’s law, bandgap at 0 K, thermal expansion coefficient, and Debye temperature was determined as a function of Bi composition. From AFM observations, bismuth islands on the surface were monitored. As bismuth composition increased, the size of the islands also increased. The intensity of Bi-induced mode at approximately 185 cm^-1^ and the broad feature in the range of 210 to 250 cm^-1^ were observed to increase with the increase of the Bi composition. Moreover, the observation of the forbidden TO mode in GaAsBi was attributed to Bi-related disorder that degraded the crystal symmetry of the structure.

## Abbreviations

AFM: atomic force microscopy; MBE: molecular beam epitaxy; PL: photoluminescence.

## Competing interests

The authors declare that they have no competing interests.

## Authors’ contributions

FS carried out the PL measurements and contributed to the writing of the article. ÖD carried out the some part of PL measurements. KK took and analyzed AFM images. AE wrote the most part of the article and carried out Raman measurements. EA carried out Raman measurements. MCA supervised the experimental work. HM, AA, and CF grew the samples. All authors read and approved the final manuscript.
